# IRF-2 inhibits cancer proliferation by promoting AMER-1 transcription in human gastric cancer

**DOI:** 10.1186/s12967-022-03275-0

**Published:** 2022-02-03

**Authors:** Yan-Jie Chen, Shu-Neng Luo, Hao Wu, Ning-Ping Zhang, Ling Dong, Tao-Tao Liu, Li Liang, Xi-Zhong Shen

**Affiliations:** 1 Department of Gastroenterology, Zhongshan Hospital Affiliated to Fudan University, NO. 180, Fenglin Road, Xuhui District, Shanghai, 200032 People’s Republic of China; 2 Department of Medical Oncology, Zhongshan Hospital Affiliated To Fudan University, NO. 180, Fenglin Road, Xuhui District, Shanghai, 200032 People’s Republic of China; 3 Cancer Center, Zhongshan Hospital Affiliated To Fudan University, NO. 180, Fenglin Road, Xuhui District, Shanghai, 200032 People’s Republic of China; 4 Center of Evidence-Based Medicine, Zhongshan Hospital Affiliated To Fudan University, NO. 180, Fenglin Road, Xuhui District, Shanghai, 200032 People’s Republic of China

**Keywords:** IRF-2, AMER-1, wnt/β-catenin signaling pathway, Gastric cancer, Prognosis

## Abstract

**Background:**

Interferon regulatory factor 2 (IRF-2) acts as an anti-oncogene in gastric cancer (GC); however, the underlying mechanism remains unknown.

**Methods:**

This study determined the expression of IRF-2 in GC tissues and adjacent non-tumor tissues using immunohistochemistry (IHC) and explored the predictive value of IRF-2 for the prognoses of GC patients. Cell function and xenograft tumor growth experiments in nude mice were performed to test tumor proliferation ability, both in vitro and in vivo. Chromatin immunoprecipitation sequencing (ChIP-Seq) assay was used to verify the direct target of IRF-2.

**Results:**

We found that IRF-2 expression was downregulated in GC tissues and was negatively correlated with the prognoses of GC patients. IRF-2 negatively affected GC cell proliferation both in vitro and in vivo. ChIP-Seq assay showed that IRF-2 could directly activate AMER-1 transcription and regulate the Wnt/β-catenin signaling pathway, which was validated using IHC, in both tissue microarray and xenografted tumor tissues, western blot analysis, and cell function experiments.

**Conclusions:**

Increased expression of IRF-2 can inhibit tumor growth and affect the prognoses of patients by directly regulating AMER-1 transcription in GC and inhibiting the Wnt/β-catenin signaling pathway.

## Background

Gastric cancer (GC) is the fifth most frequent malignancy, with 1,000,000 new cases in 2018 worldwide [1]. An estimated 679,100 new cases have developed in China each year, making GC the second most deadly form of cancer in China [2]. Although great advances have been made for its diagnosis and therapy, the prognosis of advanced GC remains poor [3]. Therefore, it is crucial to investigate the molecular pathogenesis of GC to predict the prognosis and develop potential therapeutic targets.

The interferon regulatory factor (IRF) family in humans is a transcriptional factor that can modify gene expression by directly targeting the DNA promoter sequences of target genes [4]. IRF-2 is a crucial member of the IRF family, located on chromosome 4q34.1-q35.1; IRF-2 has no tissue specificity. It reportedly plays critical roles in oncogenesis, cell apoptosis, immune regulation, and cell differentiation. Zucman-Rossi et al. [5] reported that recurrent alterations of the IRF-2 played a key role in tumorigeneses of hepatocellular carcinoma (HCC). Further studies found that IRF-2 inactivation led to impaired P53 function, making it a tumor suppressor in HCC [5, 6]. Recent research found that IRF-2 could downregulate PD-L1 promoter activity and protein levels in HCC [7]. Frequent loss of IRF-2 leads to decreased MHC class I antigen presentation and increased PD-L1 expression in cancer, resulting in immune evasion [8]. It was also found that KRAS-mediated repression of IRF-2 led to increased expression of CXCL3 and reduced expression of CXCR2. Higher IRF-2 expression leads to increased responsiveness to anti-PD-1 therapy in colorectal cancer [9].

Adenomatous polyposis coli (APC) membrane recruitment 1 (AMER-1) is a plasma membrane-associated protein containing 1135 amino acids. AMER-1 can interact with APC via three binding domains that share no obvious sequence similarities [10]. AMER-1 has been identified as a tumor suppressor that functions by regulating the Wnt/β-catenin signaling pathway. It can specifically bind phosphatidylinositol 4,5-bisphosphate, translocate to the cell membrane, and interact with key regulators of the canonical Wnt/β-catenin signaling pathway, such as components of the β-catenin destruction complex [11, 12]. The Wnt/β-catenin signaling pathway is among the key pathways involved in GC development, and can regulate the expression of various factors involved in GC differentiation, invasion, and metastasis [13]. Inhibition of the Wnt/β-catenin signaling pathway can downregulate the expressions of β-catenin, c-Myc, and CD44, and decrease the proliferation abilities of GC cells [14]. In contrast, enhanced Wnt/β-catenin activity can promote tumor formation and promote stem cell-like features in GC cells [14].

Our previous studies have found that miR-18a directly targets IRF-2 and modulates the expression of IRF-2, thus affecting the expressions of P53 and matrix metalloproteinase 1 (MMP-1) in GC [15, 16]. In this study, we further explored the functions of IRF-2, both in vitro and in vivo, and discussed its downstream pathway to explore the role of IRF-2 in GC development.

## Methods

### Patients and specimens

Tumor specimens were obtained from 72 patients with gastric adenocarcinoma (GAC) who underwent curative resection at Zhongshan Hospital of Fudan University between 2011 and 2014. The inclusion and exclusion criteria were as follows: (a) having a distinctive pathologic diagnosis of GAC. (b) Curative gastric surgical treatment with complete resection of all cancer nodule, and histological examination revealing no tumor cells on the cut surface. (c) Complete follow-up data until June 2017. (d) Suitable formalin-fixed and paraffin-embedded tissues. (e) Patients who agreed to participate in the study and signed informed consent. The GAC diagnosis was based on WHO criteria, and the tumor stage was classified according to the 7th edition of the tumor-node-metastasis (TNM) classification by the Union for International Cancer Control (UICC). Ethical approval for human subjects was obtained from the research ethics committee of Zhongshan Hospital of Fudan University. The clinical characteristics of all the patients are listed in Table 1.

**Table 1:** Correlation between IRF-2 and clinicopathologic characteristics

	Total	IRF-2 expression		*P* value
		Low (35)	High(37)	
Sex				
Male	55	28 (50.9)	27 (49.1)	0.483
Female	17	7 (41.2)	10 (58.5)	
Age(y)				
< 60	34	16 (47.1)	18 (52.9)	0.803
≥ 60	38	19 (50.0)	19 (50.0)	
Histological grade				
G1–2	32	18 (56.3)	14 (43.7)	0.460
G3	40	19 (47.5)	21 (52.5)	
Invasive depth				
Mucosa to muscularis propria	21	9 (42.9)	12 (60.6)	0.150
Adventitia to adjacent structure	51	22 (56.4)	17 (43.6)	
Lymph nodes metastasis				
≤ 2 regions	33	13 (39.4)	20 (30.77)	0.407
> 2 regions	39	31 (60.78)	20 (39.22)	
Distant metastasis				
Yes	8	6 (75.0)	2 (25.0)	0.146
No	64	29 (45.3)	35 (54.7)	
Position				
Antrum and gastric angle	32	14 (43.8)	18 (56.3)	0.460
Others	40	21 (52.5)	19 (47.5)	
Size				
< 4 cm	27	13 (48.1)	14 (51.9)	0.951
≥ 4 cm	45	22 (48.9)	23 (51.1)	
TNM stage				
I, II	28	12 (42.9)	16 (57.1)	0.436
III, IV	44	23 (52.3)	21 (47.7)	

Fisher’s exact test for distant metastasis; χ^2^ test for all other analyses

Most patients were treated with systemic chemotherapy or traditional Chinese medicine according to their clinical conditions. After treatment conclusion, follow-up was conducted every 6 months, in which patients were monitored by chest, abdomen, and pelvic enhanced CT scanning. Endoscopy was performed annually. Patients with confirmed cancer recurrence received further treatment. OS was defined as the time from the beginning of the study to patient death or study termination, while DFS was defined as the time from the beginning of the study to tumor recurrence or study termination. The 3- and 5-year disease-free survival rates were 8.33% and 6.94%, while the 3- and 5-year overall survival rates were 36.11% and 27.78%.

### Immunohistochemistry (IHC) and staining evaluation

Cancer tissue and adjacent normal tissue were formalin-fixed, paraffin-embedded, and made into tissue microarrays (TMAs) after hematoxylin and eosin (HE) staining and histopathology-guided locations. Five-micron-thick sections of TMA were deparaffinized and rehydrated, then subjected to high-temperature antigen retrieval via microwave in 0.1 M citrate solution (pH 6.0) for 15 min. The sections were incubated with mouse anti-IRF-2 antibody (Abcam, Cambridge, UK) and anti-FAM123B (Abcam, Cambridge, UK) overnight at 4 °C. The TMA sections were then incubated for 30 min with secondary antibody at room temperature and immunostained by the avidin–biotin complex technique using 3,3'-diaminobenzidine. Hematoxylin was used as a counterstain.

Two pathologists evaluated immunohistochemical staining. The interpretation of immunoreactivity was calculated by analyzing the extent and intensity of staining positivity of cells: “ ≤ 5% cell positivity” or “negative staining” = 0; “6–20% cell positivity” or “light staining” = 1; “21–50% cell positivity” or “mild staining” = 2; “ > 50% cell positivity” or “intense staining” = 3. The total score was the product of the two. The final score was determined by subtracting the score of the cancer tissue from the score of the adjacent tissue. Values greater than 2.5 were considered low expression in IRF-2, while values greater than 2 were considered low expression in AMER-1. All other values were considered high expression.

### Cell culture, transfection and virus infection

Human GC cell lines MKN-45 and SGC-7901 were obtained from the Cell Bank of Type Culture Collection of the Chinese Academy of Science, Shanghai Institute of Biochemistry and Cell Biology, Shanghai, China, and cultured in RPMI 1640 medium (HuClone, USA) supplemented with 10% fetal bovine serum (FBS, Corning, USA) at 37 °C in an incubator containing 5% CO_2_.

For the experiments utilizing overexpression, the IRF-2 full-length sequence was synthesized and subcloned into the expression vector CMV-MCS-3XFlag-PGK-Puro (Genechem, China). MKN45 cells were transfected with CMV-IRF2-3XFlag-PGK-Puro according to the manufacturer’s instructions. For the knockdown experiments, short hairpin RNA for IRF-2 (shIRF-2) was generated by Genechem (China) and inserted into the pHY-LV-KD1.4 lentiviral shRNA vector (Hanyinbt, China). SGC-7901 cells were transfected with lentiviral shIRF-2 and subjected to selection with puromycin to establish a stable cell line. Stable monoclonal cell lines with up-regulated and down-regulated IRF-2 were screened. The efficacy of overexpression and knockdown of IRF-2 were verified using real-time PCR and western blotting.

For overexpression experiments, the AMER-1 full-length sequence was synthesized and subcloned into a pcDNA3.1 vector (Genechem, China). MKN45 cells were transfected with pcDNA3.1-IRF-2 using Lipofectamine 3000 (Invitrogen, USA) according to the manufacturer’s instructions. For the knockdown experiments, SGC-7901 cells were transfected with AMER-1 siRNA according to the manufacturer’s instructions (Genechem, China).

### Protein extraction and western blot analysis

Protein extraction and western blot analysis were performed according to the standard protocols using antibodies against IRF-2 (Abcam, USA), AMER-1 (Abcam, USA), CD44 (EPITMICS, USA), c-myc (Abcam, USA), β-catenin (CST, USA), OCT-4 (Abcam, USA), and SOX-2 (Abcam, USA). β-actin (Abcam, USA) was used as the loading control.

### Real-time PCR

Total RNA was extracted from the cells and tissues using TRIzol Reagent (Invitrogen) according to the manufacturer's instructions. A total of 0.5 μg RNA from each sample was subjected to reverse transcription to obtain cDNA using a SuperScript™ III First-Strand Synthesis System Kit (Thermo Fisher Scientific). The resulting cDNA was diluted 100-fold and applied to a real-time PCR (RT-PCR) assay using a Real-time PCR System (Applied Biosystems, USA) with a SYBR Green PCR Master Mix kit (TaKaRa, Japan) following the manufacturer’s protocol. The 2^−ΔΔCt^ method was used to analyze the relative fold changes. The experiments were performed in triplicate for each data point.

The AMER-1 primers used for PCR were 5'-GGGCTGGACCCCACTGT-3′ (forward) and 5’-CTGCTCAACAGCATCTATCG-3 (reverse), while the IRF-2 primers used for PCR were 5-CGAATGCTGCCCCTATCAGA-3’ (forward) and 5’-TCCTACAACTATGATGTTCACCGT-3’ (reverse). GAPDH was used as an internal control and was detected using the following primers: 5’-AATCCCATCACCATCTTCC-3 (forward) and 5-AGTCCTTCCACGACCAA-3 (reverse).

### Detection of cell proliferation

Plate colony formation assay and 5-Ethynyl-2′-deoxyuridine (EdU) assays were conducted according to standard protocols. Briefly, 500 cells/well were seeded in 6-well plates for the plate colony formation assay. The cells were mixed and cultured for 2 weeks in a culture medium with 10% FBS. Clusters containing more than 30 cells were counted as single colonies. A Cell-Light™ EdU Apollo®488 In Vitro Imaging Kit (RiboBio, China) was used to measure cell proliferation. Images of the cells were obtained using a Nikon microscope (Nikon, Japan). All experiments were repeated three times.

Cells were seeded in a 96-well plate at a concentration of 1500 cells per well. All assays were performed in triplicate. At 1, 2, 3, 4, and 5 days after transfection, the cell proliferation assay was performed by adding 10 μl cell counting kit 8 (CCK8) solution (Beyotime, Shanghai, China) to each well. After incubation at 37 °C for 2 h, cell absorbance was measured at a 450 nm wavelength using a microplate reader (Flexstation III ROM V2.1.28, USA).

### Chromatin immunoprecipitation sequencing (ChIP-seq)

Twenty million OE-IRF2-MKN45 cells were grown and washed, then crosslinked with 1% formaldehyde for 10 min at room temperature. Crosslinking was quenched by adding glycine to a final concentration of 0.15 M for 5 min at room temperature. Crosslinked cells were washed with ice-cold PBS, the supernatant was discarded, and the pellets were flash-frozen in liquid nitrogen and stored at − 80 °C.

For each sample, 20 million fixed cells were lysed to prepare nuclear extracts. After chromatin shearing by sonication, lysates were incubated overnight at 4 °C with protein A Dynabeads coupled with 5 μg of antibody. After immunoprecipitation, the beads were recovered using a magnet and then washed. DNA was eluted and cross-links reverted at 65 °C for 4 h, then purified using the QIAGEN Kit. DNA was quantitated using the Qubit® dsDNA HS assay and a Qubit® 2.0 Fluorimeter (Invitrogen). For ChIP-Seq, 5 ng of purified ChIP DNA was used to generate the sequencing library using an NEB kit and sequenced with the Illumina HiSeq X Ten. Each sample was tested at least three times.

For ChIP-seq data analysis, FastQC software was used to evaluate the quality of the original data. The original data were then compared to the reference genome using BWA or Bowtie2 software. MACS was used for peak calling, genome location annotation of peak mining, motif analysis of peak area, and GO and KEGG enrichment analysis of the target genes.

### Luciferase assay

Then, AMER-1-wild and -mut were inserted into the pGL3 promoter vector (GenScript Co., Ltd., Nanjing, China), which was transfected into 7901 and MKN-45 cells using Lipofectamine 2000 (Invitrogen, Thermo Fisher Scientific, Inc.), along with IRF-2 overexpression vectors or NC vectors. The cells were seeded in 24-well plates. After 48 h, firefly luciferase signals and Renilla luciferase (internal reference) were detected using a dual-luciferase reporter assay kit (Promega, Madison, WI, USA.) according to the manufacturer’s protocol. All experiments were performed in triplicate.

### Xenograft tumor growth in nude mice

Ten female BALB/c nude mice (4 to 6 weeks old and weighing 18 to 20 g) were obtained from the Shanghai Experimental Animal Center (Shanghai, China). shIRF2-SGC7901 and NC-SGC7901 cells (2 × 10^6^) were harvested and injected subcutaneously into nude mice (five mice per group). Tumor growth was quantified every 2 days after tumor formation, and tumor volumes were calculated as length × width^2^ × 0.5. After about 23 days, tumors were removed when the mice were sacrificed. Tumor tissues were then formalin-fixed, paraffin-embedded, HE stained, and immunohistochemically stained to measure the expression levels of IRF-2, AMER-1, and CD44. All xenograft experiments were approved by the Animal Experiments Ethics Committee of Zhongshan Hospital of Fudan University.

### Bioinformatic investigation

The co-expression relationship between IRF-2 and AMER-1 at the mRNA level was investigated using the GEPIA database (http://gepia.cancer-pku.cn/index.html) [17]. The Spearman correlation coefficient R was calculated to describe the relationship. Samples were retrieved from the stomach cancer dataset (STAD) of The Cancer Genome Atlas (TCGA; http://tcga-data.nci.nih.gov/tcga/) database. There were 370 cases comparing the IRF-2 and AMER-1 expression levels in terms of log transformed transcripts per million (TPM) values.

### Statistical analyses

Statistical analyses were performed using SPSS (version 26.0; SPSS Inc., IL, USA) and GraphPad Prism 7 (GraphPad Software, Inc., CA, USA). Mann–Whitney test, Student’s t-test, paired t-test, χ2 test, and Fisher’s exact probability were used for comparison among groups. The Kaplan–Meier method and log-rank test were used to calculate the cumulative survival time. The prognostic value of IRF-2 was measured using univariate and multivariate analyses based on the Cox proportional hazard regression model. All tests were two-sided. *Statistical significance was set at P* < 0.05.

## Results

### The IRF-2 expression was downregulated in GC tissue and related with prognosis

**Fig. 1:**
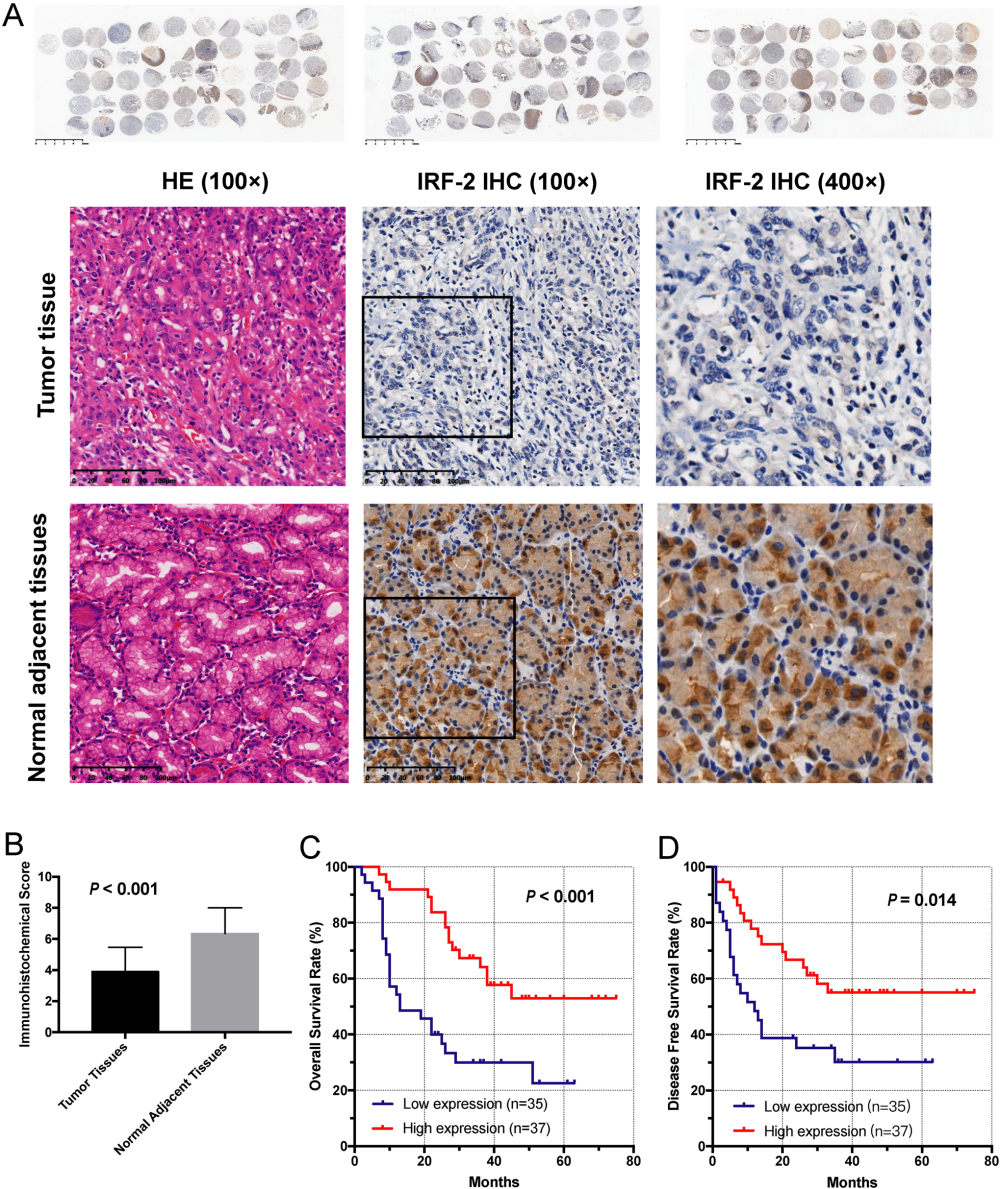
Increased IRF-2 expression is related to favorable prognosis in GC patients. **A** The IRF-2 expression level was examined in a tissue microarray containing 72 pairs of GC tissues and normal adjacent tissues by immunohistochemical analysis. **B** It was found that the expression level of IRF-2 was lower in GC tissues than in normal adjacent tissues. **C** The IRF-2 expression level was significantly correlated with patients’ OS. **D** The IRF-2 expression level was significantly correlated with patients’DFS

The expression level of IRF-2 was examined in TMAs containing 72 pairs of GC tissues and normal adjacent tissues by IHC analysis. Immunohistochemical analyses revealed that IRF-2 was mostly located in the cytoplasm and downregulated in human GC tissues compared with normal adjacent tissues (*P* < 0.001; Fig. 1A, B). The average score of IRF-2 was 3.90 ± 1.56 in GC tissues and 6.35 ± 1.65 in normal adjacent tissues.

To determine the relationship between the expression level of IRF-2 and the clinical characteristics of GC patients, we collected the patients’ data and summarized them in Table 1. There was no correlation between IRF-2 expression and clinical characteristics including age, sex, tumor size, invasive depth, lymph node metastasis, tumor position, and TNM stage (*P* > 0.05).

A Kaplan–Meier analysis and log-rank test were used to evaluate the influence of IRF-2 on survival. We found that the IRF-2 expression level was significantly positively correlated with patients’ overall survival time (OS) (*P* < 0.001, Fig. 1C) and cancer-free survival time (DFS) (*P* = 0.014, Fig. 1D), indicating that higher IRF-2 expression correlated with longer DFS and OS. Tumor size, TNM stage, invasive depth, and lymph node metastasis were unfavorable predictors for OS. Tumor size, TNM stage, invasive depth, lymph node metastasis, and distant metastases were unfavorable predictors for DFS. IRF-2 was a favorable predictor for both OS and DFS of GC (Table 2). Considering that the invasive depth, lymph node metastasis, and distant metastases were included in the TNM stage, we only included tumor size, TNM stage, and expression of IRF-2 in the multivariate analysis. This analysis found that IRF-2 was an independent prognostic factor for OS (P < 0.001) and DFS (P = 0.002).

**Table 2:** Univariate and multivariate analyses of factors associated with survival and cancer-free survival

	OS		DFS	
	Hazard ratio (95%CI)	*P* value	Hazard ratio (95%CI)	*P* value
Univariate analyses				
Sex (male vs female)	0.875 (0.417, 1.836)	0.725	1.016 (0.484, 2.131)	0.967
Age, y (< 60 vs. ≥ 60)	1.088 (0.800, 1.480)	0.589	1.050 (0.772, 1.427)	0.756
Histological grade (G1-2 vs. G3)	1.871 (0.980, 3.573)	**0.058**	1.883 (0.985,3.600)	**0.055**
Invasive depth (mucosa to muscularis propria vs. adventitia to adjacent structure)	0.434 (0.259, 0.728)	**0.002**	0.405 (0.241, 0.680)	**0.001**
Lymph nodes metastasis (≤ 2 regions vs. > 2 regions)	0.432 (0.296, 0.629)	** < 0.001**	0.422 (0.289, 0.616)	** < 0.001**
Distant metastasis (yes vs. no)	2.183 (0.912, 5.227)	0.080	2.514 (1.050, 6.018)	0.039
Position (antrum vs. others)	0.965 (0.707, 1.318)	0.824	0.945 (0.693, 1.290)	0.723
TNM stage (I, II vs III, IV)	3.775 (1.735, 8.210)	**0.001**	3.947 (1.810, 8.607)	**0.001**
Size (< 4 cm vs. ≥ 4 cm)	2.571 (1.256, 5.264)	**0.010**	2.505 (1.223, 5.131)	**0.012**
IRF-2 (positive vs. negative)	2.913 (1.538, 5.518)	**0.001**	2.517 (1.337, 4.738)	**0.004**
Multivariate analyses				
IRF-2 (positive vs. negative)	3.335 (1.736, 6.404)	** < 0.001**	2.756 (1.451, 5.234)	**0.002**
Size (< 4 cm vs. ≥ 4 cm)	1.628 (0.760, 3.485)	0.209	1.544 (0.713, 3.347)	0.271
TNM stage (I, II vs. III, IV)	3.495 (1.516, 8.058)	**0.003**	3.522 (1.509, 8.220)	**0.004**

The Cox proportional hazards regression model was used in the univariate analysis. Multivariate analysis and Cox proportional hazards regression models were used in the multivariate analysis. Variables were adopted for their prognostic significance by univariate analysis with forward stepwise selection (forward, likelihood ratio). Variables were adopted for prognostic significance using univariate analysis (*P* < 0.05). 95% CI, 95% confidence interval

### IRF-2 repressed the proliferation of GC cell

**Fig. 2:**
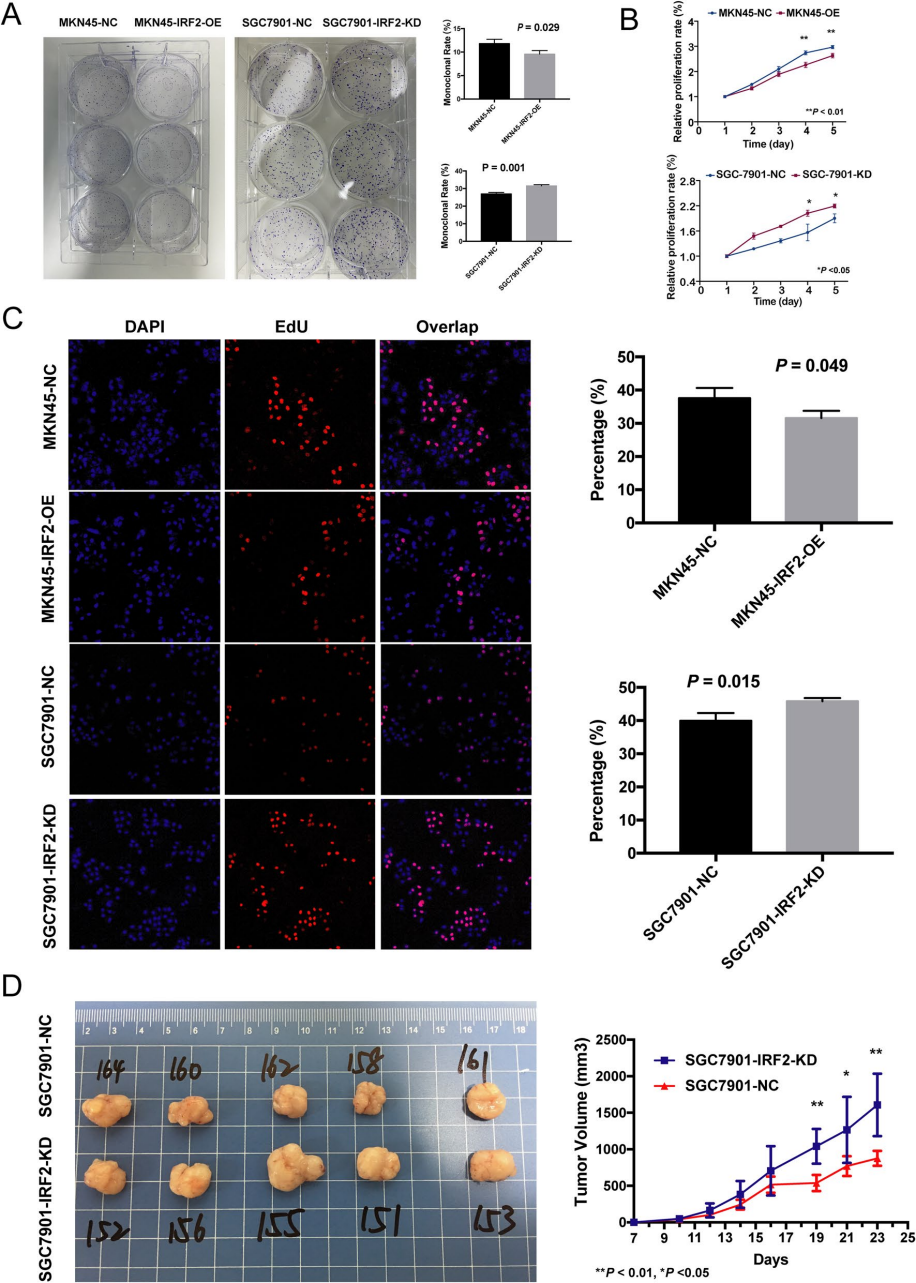
IRF-2 can affect GC cell proliferation both in vitro and in vivo. **A** Colony formation assays showed that colony formation ability was negatively correlated with IRF-2 expression. **B** CCK8 assays showed that the proliferation ability was inhibited by IRF-2. **C** EdU assays also showed that the GC cell proliferation ability was negatively correlated with IRF-2 expression. **D** In the xenograft tumor model, tumor volume increased in the IRF-2 knock down group

The stable cell lines with overexpression of IRF-2 in MKN-45 and knockdown of IRF-2 in SGC-7901 have been constructed and validated in our previous studies [16]. Colony formation assays showed that colony formation ability decreased following IRF-2 overexpression in MKN-45 cells, whereas colony formation ability increased following IRF-2 knockdown in SGC-7901 cells (Fig. 2A). Further, IRF-2 overexpression remarkably decreased the proliferation of MKN-45 cells, whereas knockdown of IRF-2 increased cell proliferation (Fig. 2B). Similarly, EdU assays also showed that IRF-2 overexpression inhibited GC cell proliferation, whereas its knockdown promoted GC cell proliferation (Fig. 2C).

We further explored whether IRF-2 affects GC growth in vivo. SGC-7901 cells stably transfected with sh-IRF-2 and empty vectors were injected into nude mice. Twenty-three days after the injection, tumors from the sh-IRF-2 group were significantly larger than those from the control group (Fig. 2D). These findings indicate that IRF-2 can negatively affect GC cell proliferation both in vitro and in vivo.

### IRF-2 directly activated AMER-1 transcription and regulated Wnt/β-catenin signaling pathway

**Fig. 3:**
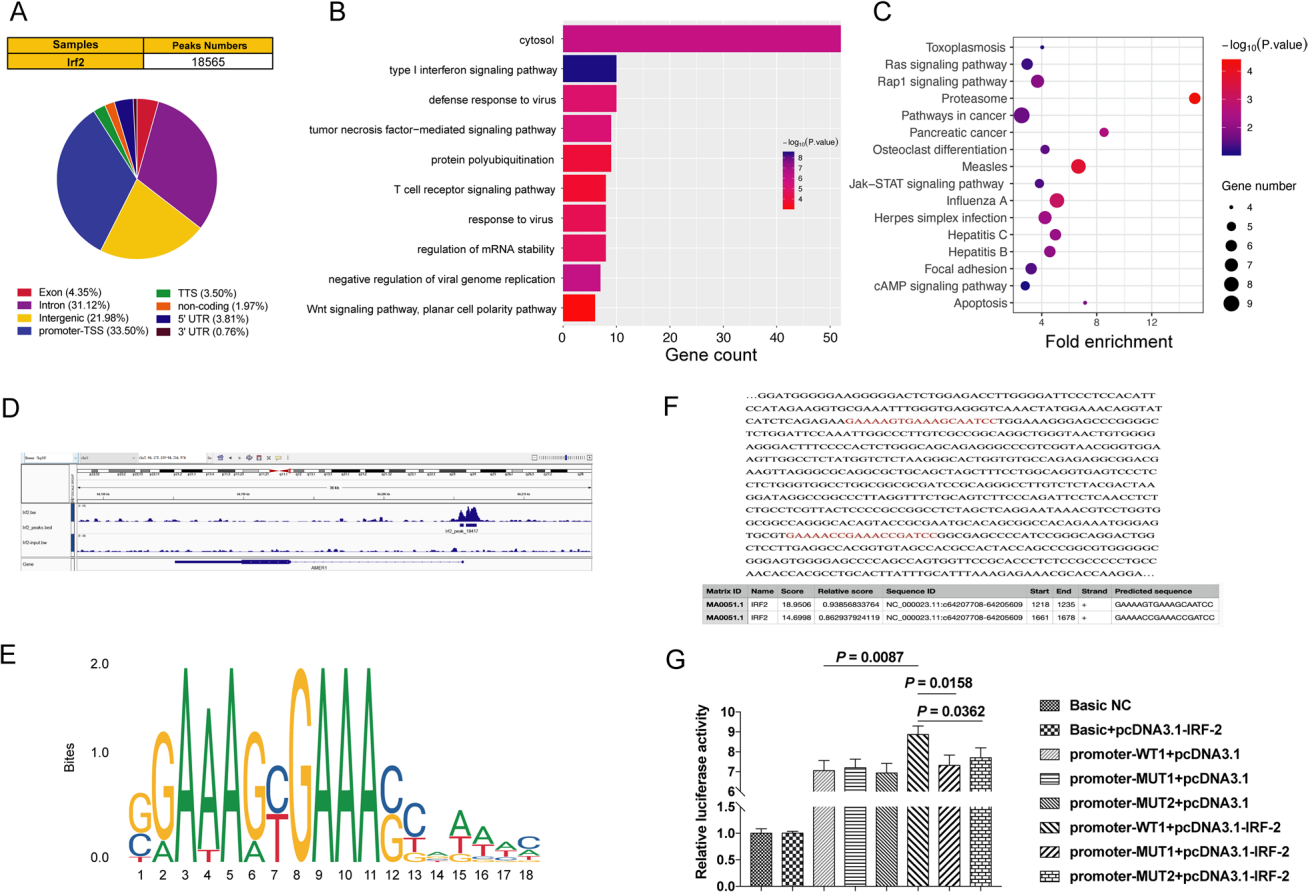
IRF-2 directly activated AMER-1 transcription and regulated the Wnt/β-catenin signaling pathway. **A** The peak information in ChIP-Seq analysis and proportion of IRF-2 binding to promoter regions. **B** Enrichment analysis of GO-Biological Precell with IRF-2 expression was shown. **C** KEGG analysis also showed that IRF-2 may affect several cancer pathways. **D** ChIP-Seq showed that IRF-2 may act on the promoter region of AMER-1. **E** Possible IRF-2 binding sites were found using the JASPAR 2020 database. **F** It was found that there were two predicted IRF-2 binding sites in the AMER-1 transcription start domain. **G** Luciferase assays for detecting luciferase activity of AMER-1-promoter-WT, AMER-1-promoter-Mut1 and AMER-1-promoter-Mut2 after IRF-2 overexpression

We applied ChIP-Seq to investigate the potential target and binding sites of IRF-2 in GC and found 18,565 peaks (Fig. 3A). GO and KEGG enrichment analysis of target genes were used to explore the signaling pathways may be affected by IRF-2. The ten pathways in which IRF-2 was most affected by GO analysis included the tumor necrosis factor-mediated signaling pathway and the Wnt**/**β-catenin signaling pathway, both of which were related to tumor development and progression (Fig. 3B). KEGG analysis also showed that IRF-2 may affect several cancer pathways (Fig. 3C). Combined with the previously reported results of microarray assays [16], we found that IRF-2 can inhibit the Wnt/β-catenin signaling pathway by directly targeting the AMER-1 transcription start domain. We also found that IRF-2 may act on the promoter region of AMER-1 to promote transcription by Chip-Seq (Fig. 3D). Possible binding sites of IRF-2 were identified using the JASPAR 2020 database (Fig. 3E) [18] and there were two predicted binding sites in the AMER-1 transcription start domain (Fig. 3F), which is consistent with our ChIP-Seq results. To determine whether IRF-2 binds to the AMER-1 promoter, we performed luciferase assays. The results showed that IRF-2 significantly upregulated the luciferase activity of AMER-1-promoter-WT, but not AMER-1-promoter-Mut1 nor AMER-1-promoter-Mut2 (Fig. 3G). This suggests that IRF-2 binds to the AMER-1 promoter in GC.

### IRF-2 promoted the expression of AMER-1

**Fig. 4:**
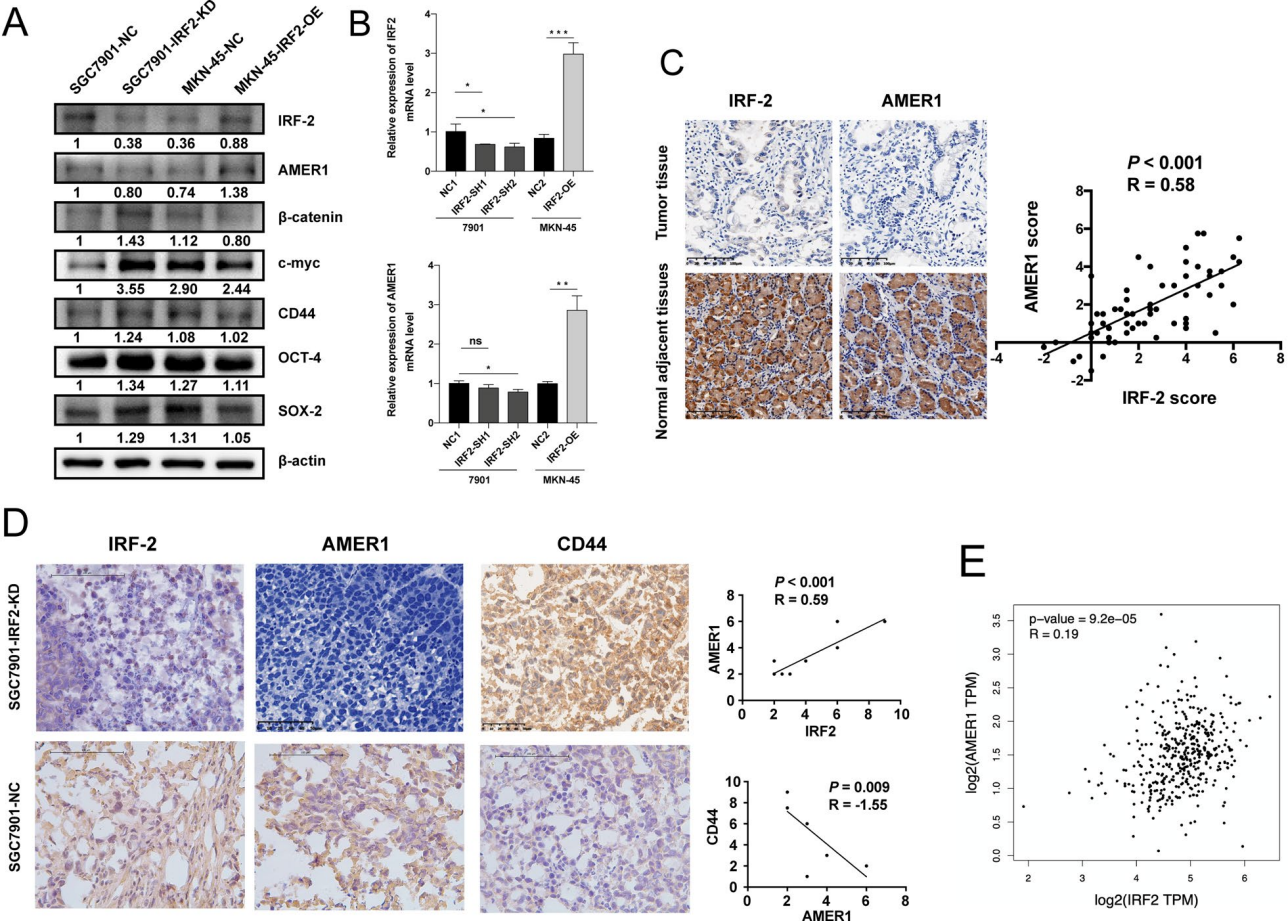
The expression level of AMER-1 was positively related to IRF-2. **A, B** Western blotting and RT-PCR verified the positive relationship between IRF-2 and AMER-1 in both protein and mRNA levels, while the Wnt/β-catenin signaling pathway was negatively correlated with the IRF-2 and AMER-1 expressions in protein levels. **C** Immunohistochemical scores showed a positive correlation between AMER-1 and IRF-2 in tissue microarray. **D** A positive correlation between AMER-1 and IRF-2 was found in xenografted tumor tissues and a significant inverse correlation was also found between the expression of AMER-1 and CD44. **E** A positive correlation was also found between the expression of IRF-2 and AMER-1 on website GEPIA. *, *P* < 0.05; **, *P* < 0.005; ***, *P* < 0.0005

We verified the AMER-1 expression and the key factors affecting the Wnt/β-catenin signaling pathway, including CD44, c-myc, and β-catenin, in lentivirus-infected cell lines. AMER-1 expression increased after IRF-2 was overexpressed, while AMER-1 expression decreased when IRF-2 was downregulated, at both the protein and mRNA levels (Fig. 4A, B). IRF-2 expression was also negatively correlated with the indices of stem cell-like features, including OCT-4, SOX-2, CD44, and c-myc (Fig. 4A). To further evaluate the relationship between IRF-2 and AMER-1 in GAC patients and xenografted tumor tissues in nude mice, we examined the expression levels of AMER-1 by immunohistochemical assay, using anti-AMER-1 antibody in the same TMA specimens and xenografted tumor tissues. The immunohistochemical scores showed positive correlations between AMER-1 and IRF-2 scores, both in TMA specimens (r = 0.58, P < 0.001; Fig. 4C) and xenografted tumor tissues (r = 0.59, P < 0.001; Fig. 4D). A significant inverse correlation was also found between AMER-1 expression and CD44 (*r* = − 1.55, *P* = 0.009; Fig. 4D). A positive correlation was also found between the expressions of IRF-2 and AMER-1 on the GEPIA (*r* = 0.19, *P* < 0.001; Fig. 4E).

### IRF-2 inhibiting Wnt/β-catenin Signaling Pathway was dependent on AMER-1

**Fig. 5:**
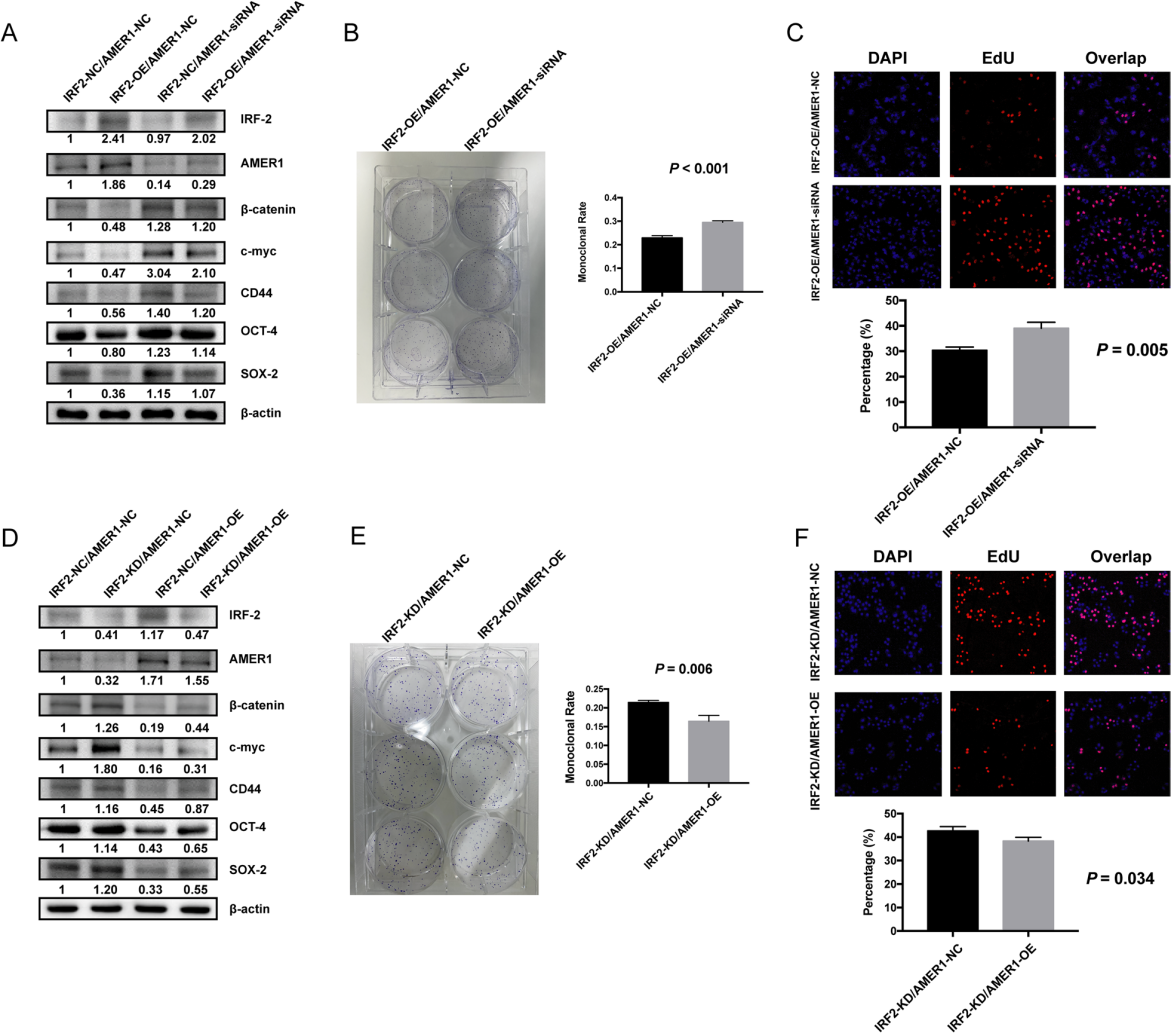
IRF-2 inhibiting the Wnt/β-catenin signaling pathway depends on the regulation of AMER-1. **A, D** Western blotting verified that the negative regulatory effect of IRF-2 on Wnt/β-catenin pathway was dependent on AMER-1. **B, E** Colony formation ability was increased when AMER-1 was knocked down, even when IRF-2 was overexpressed. The opposite result was found when AMER-1 was over-expressed even if the IRF-2 was knocked down. **C, F** EdU assays also showed that the cell proliferation ability depended on AMER-1 regulation

To determine whether IRF-2 regulated the expression of the Wnt/β-catenin signaling pathway by targeting AMER-1, we knocked down the expression of AMER-1 in MKN-45 cells with or without overexpressed IRF-2. We found that key molecules in the Wnt/β-catenin signaling pathway and stem cell-like features were upregulated when AMER-1 was downregulated, regardless of the expression of IRF-2 (Fig. 5A). Similar results were also observed in the cytofunctional experiments. Knocking down the expression of AMER-1 led to increased colony formation ability (Fig. 5B) and promoted GC cell proliferation (Fig. 5C), despite overexpression of IRF-2. Similarly, inhibition of the Wnt/β-catenin signaling pathway was observed, in both western blot analyses and cytofunctional experiments, when AMER-1 was upregulated, regardless of IRF-2 expression (Fig. 5D–F). All the results indicated that the ability of IRF-2 to inhibit the Wnt/β-catenin signaling pathway was dependent on AMER-1 regulation.

## Discussion

GC is a common malignancy, with a large proportion of cases reported in East Asia. Although clinical diagnosis and treatment techniques are improving, the prognosis for gastric cancer remains poor; the 5-year survival rate is approximately 18% [1]. Therefore, it is very important to screen and study molecules that can predict the prognoses of GC patients. In this study, we confirmed in clinical samples that IRF-2 expression was lower in GC tissues than in normal tissues, and that its expression was correlated with prognosis. The IRF-2 expression level is an independent risk factor for GC patient prognosis. The results of this study are consistent with our previous results [16]. The role of IRF-2 varies among different tumor types. IRF-2 promotes malignant behaviors including glycolysis, cell proliferation, and cell cycle arrest in nasopharyngeal cancer [19]; IRF-2 can also induce cell apoptosis and repress cell proliferation and migration ability in non-small cell lung cancer [20, 21]. Li reported that microRNA-520c contributed to tumorigenesis and metastasis by downregulating IRF-2 expression at the mRNA level [22], which was in agreement with our previous findings. However, most studies have focused on the upstream regulatory mechanism of IRF-2 expression, while the downstream molecular mechanism by which IRF-2 exerts its function is not fully understood.

Ghafar investigated serum NRP-1 levels in patients with hepatocellular carcinoma and evaluated the diagnostic value [23] of NRP-1 as a serological marker. Habib identified a relationship between circulating microRNA-150 levels and imatinib response in patients with chronic myeloid leukemia and believed that microRNA-150 could be used as a predictive marker in clinical settings [24]. In this study, we first investigated the differential expression of IRF-2 in GC tissue samples and analyzed its prognostic value GC patients. We confirmed that IRF-2 was downregulated in GC and that patients with high IRF-2 expression levels had better survival times compared to those with low IRF-2 expression levels.

**Fig. 6:**
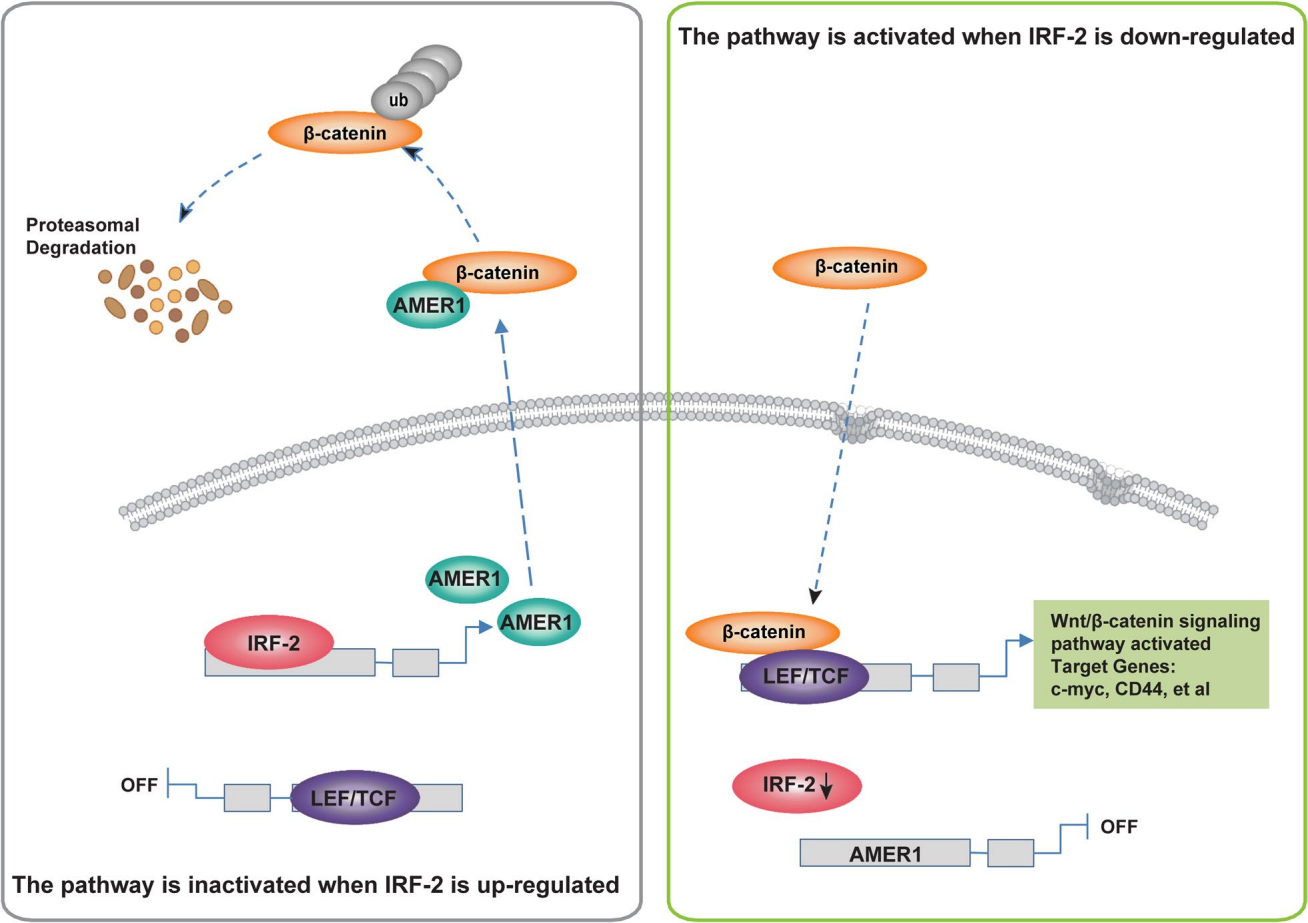
Signal mechanism of IRF-2 adversely affected the Wnt/β-catenin signaling pathway by regulating AMER-1 expression

We then performed in vitro and in vivo assays, which validated that overexpression of IRF-2 in GC cell lines resulted in both the capability of colony formation and cell proliferation as well as the upregulation of AMER-1 protein levels and activation of the Wnt/β-catenin signaling pathway; IRF-2 knockdown resulted in the opposite trend. The Wnt signaling pathway has been recognized as a promoter in many types of cancer, and its activity is controlled by a series of tumor suppressors [25, 26]. Ghafar suggested that the Wnt cascade is activated by MTDH, and exerts oncogenic functions in colorectal cancer [27]. Yang et al. reported that APC, a negative regulator of the canonical Wnt/β-catenin pathway, was upregulated by LINC01133 in a manner inhibiting proliferation and migration of GC cells [28]. Dai determined that circFGD4 enhanced APC expression by sponging miR-532-3p to repress Wnt activation and GC development [29], while APC and Wnt could also be regulated by other miRNAs such as miR-192 and miR-215 [30]. Since AMER-1 acts as a scaffold protein that recruits APC to consist of the β-catenin destruction complex, we believe that IRF-2 functions as a tumor suppressor gene in GC and that the axis of IRF-2-AMER-1/β-catenin plays an important role in the phenotype of cell proliferation (Fig. 6).

In contrast, transcription factors are involved in tumor progression. El-Guindy indicated that OCT4 is highly expressed in GC and is associated with worse survival outcomes [31]. Although IRF family is involved in immune responses and human autoimmune diseases as it is conventionally regarded as the transcriptional regulator of interferon expression [32, 33], we agree to the point that IRFs also correlated with tumor immunity or tumorigenesis [34]. Therefore, we focused on the function of IRF-2 in GC from the perspective of transcriptional regulation. We performed chromatin immunoprecipitation combined with DNA sequencing to map DNA binding by IRF-2, then further verified the binding site of the AMER-1 and IRF-2 transcription start domains by luciferase reporter assays. Qi demonstrated that IRF-2 is a transcription factor of CENP-N that promotes CENP-N expression and activates the AKT cascade in nasopharyngeal cancer [19], while Liao proved that IRF-2 binds to the DNA promoter region of CXCL3 and blocks its transcription in colorectal cancer [9]. In this study, we verified the regulatory relationship between IRF-2 and AMER-1 based on the screening results of the DNA–protein interaction. To the best of our knowledge, this is the first study to investigate the role of IRF-2 in transcription regulation in GC.

## Conclusions

Based on these results, we consider IRF-2 to be a prognostic biomarker in clinical settings because its high expression indicates relatively long survival times for GC patients. Our work also revealed that IRF-2 repressed tumorigenesis by modulating the Wnt/β-catenin signaling pathway by directly upregulating AMER-1 at the transcript level.

## Data Availability

The data that support the findings of this study are included in the main body of the manuscript and supplemental data.

## Abbreviations

IRF-2: Interferon regulatory factor 2
IHC: Immunohistochemistry
ChIP-Seq: Chromatin Immunoprecipitation assay
GC: Gastric cancer
HCC: Hepatocellular carcinoma
APC: Adenomatous polyposis coli
AMER-1: APC membrane recruitment 1
GAC: Gastric adenocarcinoma
TNM: Tumor-node-metastasis
UICC: International Union Contra Cancrum
TMAs: Tissue microarrays
HE: Hematoxylin and eosin
shIRF-2: Short hairpin RNA for IRF-2
EdU: 5‐Ethynyl‐2′‐deoxyuridine
OS: Overall survival time
DFS: Disease free survival time
ICI: Immune checkpoint inhibitors
anti-PD-1/L1: Anti-programmed death-1/anti-programmed death ligand-1
anti-CTLA4: Anti-cytotoxic T-lymphocyte-associated protein 4
MMP-1: Matrix Metalloproteinases 1
